# 1109. Pharmacokinetics and Exposure of Cefepime in Critically Ill Patients Receiving Extracorporeal Membrane Oxygenation (ECMO)

**DOI:** 10.1093/ofid/ofab466.1303

**Published:** 2021-12-04

**Authors:** Abigail K Kois, Jason A Gluck, David P Nicolau, Joseph L Kuti

**Affiliations:** 1 Hartford Hospital, Hartford, Connecticut; 2 Hartford Healthcare, Hartford, Connecticut; 3 Center for Anti-Infective Research and Development, Hartford Hospital, Hartford, Connecticut

## Abstract

**Background:**

ECMO is a life-saving tool utilized in critically ill patients that require respiratory and/or cardiac support. ECMO may also affect the pharmacokinetics (PK) of certain medications, including some antibiotics. Cefepime is a widely used antibiotic in this population due to its broad spectrum activity but limited data are available to guide dosing in patients requiring ECMO.

**Methods:**

This was a prospective, single-center study of 6 critically ill adult patients requiring ECMO and receiving cefepime 2g q8h as a 3h infusion. After obtaining informed consent, 4-6 blood samples within the dosing interval were collected to determine cefepime concentrations. Population PK was conducted in Pmetrics using R. Final MAP Bayesian parameter estimates were used to simulate free time above MIC (%ƒT >MIC) for various cefepime dosing regimens. The target pharmacodynamic exposure was 70% *f*T >MIC.

**Results:**

Patients were between 31-62 years old; 4/6 (66.7%) were on veno-venous (VV) ECMO and 2 veno-arterial (VA) ECMO. Two patients required continuous venovenous hemodiafiltration (CVVHDF) while the other 4 had a CrCL between 92-199 ml/min. A two compartment model fitted the data better than a one compartment model. Median (range) final population PK parameters were: clearance (CL), 9.8 L/h (7.6-33.1); volume of central compartment (V_C_ ), 6.9 L (4.7-49.8); and intercompartment transfer constants (k_12_), 2.04 h^-1^ (1.48-2.29); and k_21_, 1.49 h^-1^ (0.75-1.71). The 2g q8h (3h infusion) regimen resulted in target exposure in all patients up to an MIC of 8 mg/L (the susceptibility breakpoint for *Pseudomonas*), with 5/6 patients achieving this at 16 mg/L. A standard 2g q12h (0.5h infusion) regimen would have resulted in 5/6 patients achieving 70% ƒT >MIC at 8 mg/L and 1/6 at 16 mg/L.

**Conclusion:**

These are the first data describing cefepime PK and exposure attainment in critically ill patients receiving ECMO. Cefepime 2g q8h (3h infusion) achieved target pharmacodynamic exposure up to the susceptibility breakpoint of 8 mg/L in all 6 patients, including 2 with concomitant CVVHDF. Additional studies are warranted to define cefepime PK in patients on ECMO across a robust range of CrCL to guide dosing.

**Disclosures:**

**David P. Nicolau, PharmD**, **Abbvie, Cepheid, Merck, Paratek, Pfizer, Wockhardt, Shionogi, Tetraphase** (Other Financial or Material Support, I have been a consultant, speakers bureau member, or have received research funding from the above listed companies.) **Joseph L. Kuti, PharmD**, **Allergan** (Speaker’s Bureau)**BioMérieux** (Consultant, Research Grant or Support, Speaker’s Bureau)**Contrafect** (Scientific Research Study Investigator)**GSK** (Consultant)**Merck** (Research Grant or Support)**Paratek** (Speaker’s Bureau)**Roche Diagnostics** (Research Grant or Support)**Shionogi** (Research Grant or Support)**Summit** (Scientific Research Study Investigator)

